# Vitamin D Binding Protein Isoforms and Apolipoprotein E in Cerebrospinal Fluid as Prognostic Biomarkers of Multiple Sclerosis

**DOI:** 10.1371/journal.pone.0129291

**Published:** 2015-06-05

**Authors:** Simona Perga, Alessandra Giuliano Albo, Katarzyna Lis, Nicoletta Minari, Sara Falvo, Fabiana Marnetto, Marzia Caldano, Raffaella Reviglione, Paola Berchialla, Marco A. Capobianco, Maria Malentacchi, Davide Corpillo, Antonio Bertolotto

**Affiliations:** 1 Neurology Unit 2 & Regional Referral Multiple Sclerosis Centre (CReSM), San Luigi University Hospital, Orbassano, Italy; 2 ABLE Biosciences, Bioindustry Park Silvano Fumero SpA, Colleretto Giacosa, Italy; 3 LIMA, Bioindustry Park Silvano Fumero SpA, Colleretto Giacosa, Italy; 4 Neuroscience Institute Cavalieri Ottolenghi (NICO), c/o San Luigi University Hospital, Orbassano, Italy; 5 Department of Clinical and Biological Sciences, University of Turin, Turin, Italy; University of Münster, GERMANY

## Abstract

**Background:**

Multiple sclerosis (MS) is a multifactorial autoimmune disease of the central nervous system with a heterogeneous and unpredictable course. To date there are no prognostic biomarkers even if they would be extremely useful for early patient intervention with personalized therapies. In this context, the analysis of inter-individual differences in cerebrospinal fluid (CSF) proteome may lead to the discovery of biological markers that are able to distinguish the various clinical forms at diagnosis.

**Methods:**

To this aim, a two dimensional electrophoresis (2-DE) study was carried out on individual CSF samples from 24 untreated women who underwent lumbar puncture (LP) for suspected MS. The patients were clinically monitored for 5 years and then classified according to the degree of disease aggressiveness and the disease-modifying therapies prescribed during follow up.

**Results:**

The hierarchical cluster analysis of 2-DE dataset revealed three protein spots which were identified by means of mass spectrometry as Apolipoprotein E (ApoE) and two isoforms of vitamin D binding protein (DBP). These three protein spots enabled us to subdivide the patients into subgroups correlated with clinical classification (MS aggressive forms identification: 80%). In particular, we observed an opposite trend of values for the two protein spots corresponding to different DBP isoforms suggesting a role of a post-translational modification rather than the total protein content in patient categorization.

**Conclusions:**

These findings proved to be very interesting and innovative and may be developed as new candidate prognostic biomarkers of MS aggressiveness, if confirmed.

## Introduction

Multiple sclerosis (MS) is a complex pathology, presumably of autoimmune origin, of the central nervous system (CNS). Its clinical course is unpredictable and varies greatly from patient to patient, ranging from very aggressive to benign forms, stationary for decades even if not treated with specific therapies. Unfortunately, to date there are no biological markers that are capable of distinguishing the various clinical forms at diagnosis. Biomarkers of this kind are essential for choosing the most suitable and timely treatment. In fact, benign patients are clinically stable without treatment, while an early and effective therapy for aggressive-relapsing forms could significantly affect the course of the disease, thus causing less permanent damage and leading to better quality of life [1].

CSF represents a unique repository of substances secreted by the CNS, demonstrating the presence and the progression of neurological diseases. Therefore, the study of inter-individual differences in the CSF proteome may lead to the discovery of innovative markers that would be useful for prognosis [2].

Although proteomic-based approaches are excellent techniques for biomarker investigation, they have not yet given reliable results in CSF biomarker studies [3] due to technique variability and pre-analytical factors such as sample collection, patient heterogeneity and difficulties in defining and selecting patients and control groups [4, 5]. Moreover, one of the main problems observed when carrying out CSF proteomic studies is the small amount of CSF obtained from each subject. In fact, most of the CSF proteomic data were obtained from “pooled” samples [6, 7, 8, 9] and not from single-patient CSF analysis [10, 11, 12, 13]: CSF pooling may minimize the potential inter-individual differences of CSF protein content among single patients which are not associated with the underlying disease. These variations in individual CSF protein content may be responsible for the extremely controversial results of previous CSF proteome studies in neurological diseases even when using the same technical approach. On the other hand, CSF pooling may also hinder the detection of markers of various subtypes of neurological diseases linked to potentially different pathomechanisms [10].

Therefore, in order to overcome these artefacts, a two dimensional electrophoresis (2-DE) study with all the procedures and methodological steps standardized as described by Teunissen *et al*, 2009 [4] was carried out.

Furthemore, we analyzed the single patient CSF proteome(s) by performing at least three technical replicates thanks to the availability of a large amount of CSF from each patient, obtained by means of a new drawing procedure developed by neurologists at the Regional Referral Multiple Sclerosis Centre (CReSM) [14].

In order to avoid bias related to treatment [5] and gender, we enrolled a group of 24 untreated women undergoing lumbar puncture (LP) for suspected MS and we then monitored them for 5 years.

We decided not to include a control group for various reasons: the difficulty in obtaining CSF samples from healthy controls and the confusion concerning the recently-defined selection criteria for adequate neurological controls [5]. The aim of this study was to identify biomarkers with prognostic rather than diagnostic value.

By means of the hierarchical cluster analysis of proteomic data and correlation with clinical parameters, we identified potential biomarkers that were capable of stratifying MS patients according to the clinical aggressiveness of the disease.

## Materials and Methods

### Standard protocol approval, registration and patient consent

The study was approved by the Piedmont and San Luigi Hospital Ethics Committee and all the partecipants signed a written informed consent.

### Patients and CSF collection

The 24 cases enrolled in the study were selected according to the following clinical laboratory inclusion criteria: a) female gender; b) clinical signs or symptoms suggesting MS [15]; c) never treated with DMT; d) subjected to LP between January 2008 and April 2009; e) availability of more than 15 ml of CSF.

The LP was performed by neurologists at the Neurology Unit 2—CReSM of the San Luigi University Hospital, where patients were clinically monitored for the following 5 years. CSF samples were collected during the remitting phase and from patients who were not undergoing therapy.

The mean age at the the moment of the LP was 36.5 ± 10.8 years (SD, standard deviation), ranging from 17 to 61 years of age. Detailed clinical patient information is summarized in Table 1. Follow-up data of patient MS 41 are not available since this patient was lost to follow-up. The LP was performed in the L4-L5 intervertebral space following a procedure that causes very low percentages of post-LP headache and provides up to 20 ml of CSF [14]. The LP was performed using a 25 gauge Sprotte needle. This procedure takes longer than the traditional LP performed with 20 or 22 gauge needles because it requires an introducer and the aspiration of the CSF, but it reduces the risk of post-dural puncture headache to less than 2% and allows the collection of 20 ml of CSF.

**Table 1:** Clinical data of patients enrolled in the study.

Patients	Gender	EDSS at LP time	ΔEDSS at 5 years fu	Relapse N° at 5 years fu	RR at 5 years fu	DMT at 2 years fu	DMT at 5 years fu	Clinical class. at 2 years fu	Clinical class. at 5 years fu	Final diagnosis
**MS25**	F	6	0	0	0	none	none	PPMS	PPMS	PPMS
**MS26**	F	1	1*	1*	0.2*	I	death	M	death	RRMS
**MS27**	F	1	2.5	4	0.8	II	II	H	H	RRMS
**MS28**	F	1.5	5	3	0.6	I	II	M	H	RRMS
**MS29**	F	6	0	3	0.6	II	II	H	H	RRMS
**MS30**	F	1	0	1	0.2	I	I	M	M	RRMS
**MS31**	F	0	1	3	0.6	none	I	L	M	RRMS
**MS32**	F	1.5	0,5	1	0.2	I	I	M	M	RRMS
**MS33**	F	0	1	2	0.4	I	I	M	M	RRMS
**MS34**	F	0	0	0	0	none	none	L	L	RRMS
**MS35**	F	1	1	2	0.4	I	I	M	M	RRMS
**MS36**	F	1	0	0	0	none	none	L	L	RRMS
**MS37**	F	0	1	0	0	I	I	M	M	RRMS
**MS38**	F	1	0	2	0.4	I	I	M	M	RRMS
**MS39**	F	1.5	0	0	0	none	none	L (B)	L (B)	Benign MS
**MS40**	F	1	0	1	0	none	none	L (B)	L (B)	Benign MS
**MS41**	F	3.5	0**	lost	lost	***	lost	lost	lost	RRMS**
**MS42**	F	0	1	1	0.2	I	I	M	M	RRMS
**MS43**	F	4	0	?	?	I	I	M	M	RRMS
**MS44**	F	2	0	3	0.6	II	II	H	H	RRMS
**MS45**	F	0	0	0	0	none	none	L	L	RRMS
**MS46**	F	0	0	2	0.4	I	I	M	M	RRMS
**MS47**	F	2	0	4	0.8	II	II	H	H	RRMS
**MS48**	F	no MS	no MS	no MS	no MS	no MS	no MS	no MS	no MS	Somatoform disorder

F = female; LP = lumbar puncture time; EDSS = Expanded Disability Status Scale; ΔEDSS = difference between EDSS assessment at the diagnosis after 5 years of follow-up; fu = follow-up; RR = relapse-rate; DMT = disease-modifying treatments, indicated as: first line (I), second line (II) pharmacological treatments or absence of therapy (none). Clinical class. = clinical classification according: H (high) = highly aggressive disease, second-line therapy; M (moderate) = moderately aggressive disease, first-line therapy; L (low) = mildly aggressive disease; L (B) = “benign” disease, characterized by absence or progression no therapy. PPMS = primary progress multiple sclerosis; RRMS = relapsing remitting multiple sclerosis

* data available at 2-year follow-up

** data available at 1-year follow-up

*** no therapy prescribed at diagnosis.

CSF collection, storage and bio-banking were performed according to the consensus protocol described by C. Teunissen *et al*., [4]. CSF was collected in polypropylene tubes. Protease inhibitors (Leupeptin, Cat. No. L2884; Pepstatin A, Cat. No. P4265; Aprotinin, Cat. No. A4529, all bought from Sigma Aldrich, Missouri, USA) were added and CSF was centrifuged at 1,000 x g at 4°C for 10 min in order to remove cells and insoluble material and finally stored at –80°C until analysis. CSF samples were only included in the 2-DE study if more than 15 ml of CSF were available and if they contained less than 5 erythrocytes per μl. Detailed CSF biochemical data are reported in Table 2.

**Table 2:** Biochemical features of CSF in individual patients enrolled in the study.

Patients	CSF protein (mg/dl)	Serum protein (mg/dl)	CSF IgG (mg/dl)	Serum IgG (mg/dl)	CSF Albumin (mg/dl)	Oligoclonal Bands	IgG Index	Cells/ml
**MS 25**	20	683	0.585	443.7	8.7	-	0.44	3
**MS 26**	34	1190	3.52	345.6	15.7	+	0.65	5
**MS 27**	36	1467	11.5	378.5	14.4	+	2.1	28
**MS 28**	72	909	7.32	382.7	x	+	0.5	20
**MS 29**	30	1562	5.08	494.5	16.9	+	1	16
**MS 30**	22	1475	3.72	436.4	9.3	+	1.18	20
**MS 31**	35	1170	18.7	548.4	13.4	+	6.5	45
**MS 32**	36	634	3.38	360.1	13.7	+	1.4	8
**MS 33**	35	790	6.21	443.8	24.1	+	1.4	38
**MS 34**	33	920	7.22	448.6	23.6	+	1.5	16
**MS 35**	25	892	2.75	437.8	21	+	0.6	3
**MS 36**	38	1002	7.67	325.8	17.4	+	1.4	5
**MS 37**	29	1072	3.39	338.7	19.2	+	0.6	3
**MS 38**	21	988	3.32	362.2	12.6	+	0.1	8
**MS 39**	59	903	4.96	349	39.1	+	0.5	3
**MS 40**	49	1133	3.03	522.7	22.2	-	0.63	3
**MS 41**	36	1241	3.51	297.5	23.5	-	0.4	3
**MS 42**	32	1023	1.62	397.8	23.5	-	0.3	2
**MS 43**	46	1463	6.74	383.8	27.6	+	0.6	8
**MS 44**	40	1310	2.27	372.3	33.5	+	0.2	2
**MS 45**	43	1052	3.66	402	33.1	-	0.42	2
**MS 46**	35	955	4.7	346.1	20.7	+	0.82	8
**MS 47**	29	1103	4.39	432.7	17.3	+	1	6
**MS 48**	31	644	1.61	334.5	23.6	-	0.4	2

### CSF sample preparation

All phases related to sample preparation, 2-DE and statistical analysis were performed by researchers blindly as regards the clinical data-set in order to avoid bias in the results.

Each CSF sample was concentrated up to 20-fold and desalted by means of Ultrafree MC cartridges with a 5 kDa cut-off (Millipore, Bedford, USA). Following this step, the protein concentration was determined with the Bradford method using bovine serum albumin as protein standard.

### Two-dimensional electrophoresis

2-DE experiments were carried out in triplicates according to Robotti *et al*. [16] and performed after conclusion of the CSF sample collection. An amount of 100 μg total proteins from each sample was loaded for each 2-DE gel.

### Image analysis

The gels were stained with Ruby Sypro (Invitrogen, Eugene, USA) according to the manufacturer’s instructions and scanned with a ProXPRESS 2D CCD camera (Perkin Elmer, Waltham, USA). Image analysis was performed with the Image Master 2D Platinum 5.0 software package (GE Healthcare, Uppsala, Sweden). Spot detection and gel matching were carried out automatically and checked manually. For the quantitative analysis, the volume (i.e. the area of the spots multiplied by intensity) of each spot was normalized to the total volume of matched spots in the gel (%Vol). In order to visualize phosphorylated proteins, the gels were stained with Pro-Q Diamond (Invitrogen, Eugene, OR) [17] prior to incubation with Sypro.

### Data elaboration: hierarchical clustering, statistical and discriminant analysis

**Fig 1:**
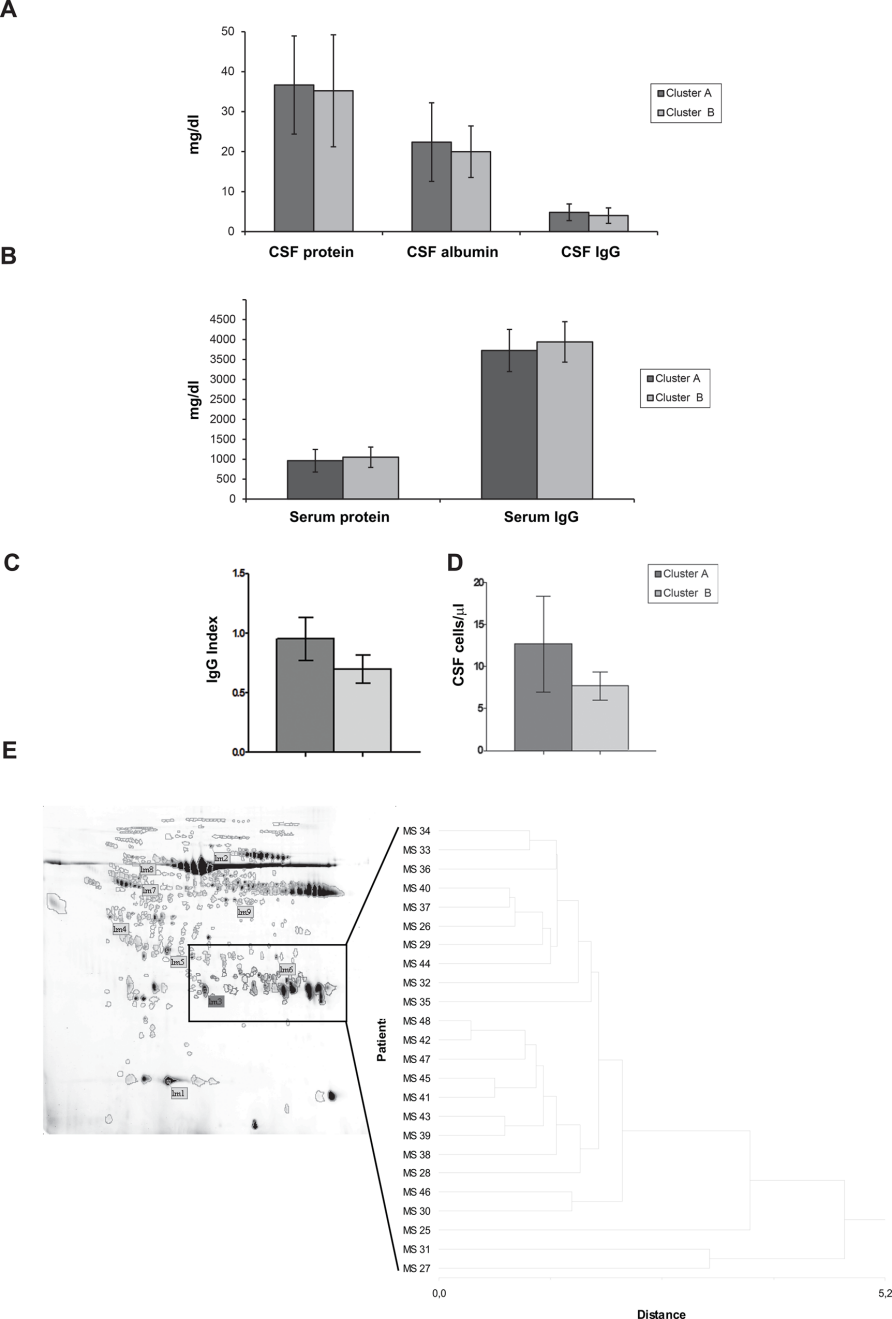
Biochemical features of CSF of patients in clusters A and Cluster B. Column bar graphs representing the mean value ± SD of total protein, albumin and Immunoglobulin (IgG) content in the CSF (A) and serum (B), Ig Index (C) and number of cells/μl in the CSF (D) of samples from patients included in cluster A and B. The statistical analysis showed that there are no statistical differences between clusters A and B for the analysed parameters. (E) The hierarchical cluster analysis based on spot representing IgG proteins highlighted patients MS25, MS31 and MS27 as outliers.

Hierarchical clustering and linear discriminant analysis were performed with StatisticXL Version 1.x software (CustomCD, http://www.statisticxl.com). The unweighted pair group method with arithmetic mean (UPGMA) was selected as the clustering method and Euclidean distance was selected as the similarity measure. The statistical analysis (MannWhitney’s test, threshold: P<0.05, performed using GraphPad Instat version 3.00) and fold-change criteria (threshold: >1.5) were applied in order to detect spots which varied in their %Vol in the identified clusters. MannWhitney’s test was also applied for evaluating the differences between cluster A and B related to age and total protein and albumin concentration in either CSF or serum (Fig 1A and 1B and Table 2), Ig Index or cell number. Predictive values were derived from the observed prevalence of the various forms of the disease. The differences in proportion of the various aggressive forms of MS across the clusters were assessed by χ^2^ test.

### Protein identification by mass spectrometry

Protein spots were excised from the 2-DE gels and analyzed by means of the HP 1100 nanoLC system together with a XCT-Plus nanospray-ion trap mass spectrometer (Agilent) as previously described [18]. Mass spectrometry data were fed into the Mascot search algorithm in order to search against the NCBI non-redundant database NCBI number 20150213 (http://www.Matrixscience.com). Hits with a probability-based Mowse score higher than 47 were considered significant (p < 0.05).

### 2-DE Western blotting

2-DE gels were transferred to nitrocellulose membranes with the semidry transfer method. The membranes, saturated with 5% BSA, were incubated over-night with specific primary antibodies: anti-ApoE (sc-13521, Santa Cruz Biotechnology, Heidelberg, Germany) used at 1:5000 dilution and anti-DBP (SAB2501100) Sigma Aldrich, Missouri, USA) used at 1:500 dilution, followed by specific HRP-conjugated secondary antibodies. The membranes were developed with an ECL Western Blotting substrate kit (Thermo Scientific, Rockford, IL USA).

### Definition of the clinical criteria for classification

Clinical classification was performed by the neurologists of CReSM according to the degree of disease aggressiveness, based on the type of disease-modifying treatment (DMT) used or proposed at the end of the 5-year follow-up. The neurologists were not aware of the aim of the study and were not involved in any phase of data elaboration. An intermediate clinical evaluation was carried out 2 years later. The DMT was chosen by the neurologists according to the indications of the European Medicine Agency (EMA) (http://www.ema.europa.eu/ema/) and the Italian Medicine Agency (AIFA) (http://www.agenziafarmaco.gov.it/en). DMT are subdivided in first and second-line drugs. First-line treatments include Interferon β (IFNβ) and Glatiramer acetate while second-line treatments include Natalizumab (NAT) and Finglimod (FIN). Second-line drugs can be used for non-responders to first-line drugs, when they have at least two relapses within one year of treatment. Patients may also be prescribed NAT or FIN as first DMT if they have an aggressive disease characterized by two relapses in the previous year and Expanded Disability Status Scale (EDSS) ≥2 [19]. All the drugs are available for all patients without restriction since the Italian National Health System offers all Italian citizens therapeutic options which are free for chronic and oncological diseases.

According to the above-mentioned criteria, highly (H), moderately (M) and “benign” or scarcely (L) aggressive forms were observed (Table 1). The H group included patients treated with second-line therapies (NAT) as first treatment or changed from a first-line treatment during the 5-year follow-up; the M group included patients treated with first-line therapies with one or less relapses per year; the L group consisted of untreated patients due to no or low disease activity and no progression of disability (Table 1). This group also included the “benign” forms (L-B) defined as patients with a stable disease over a 10-year period without any pharmacological therapy.

### Data Availability

Raw data regarding CSF 2-DE maps, spots %Vol of each replicate used for variability testing, spots %Vol used for final cluster analysis, Mass spectrometry protein identification and CSF 2-DE maps are available as Supporting Information files (S1 Dataset, S2 Dataset, S3 Dataset and S1 Images respectively).

## Results

### Pre-analytical phase: CSF collection, preparation and 2-DE analysis

2-DE technology was used for studying the proteome pattern of the CSF samples collected from a population of untreated women with MS. 2-DE was carried out on the whole protein samples without depleting high-abundant proteins such as albumin. We chose this strategy considering that albumin removal may lead to the loss of the potential biomarkers it carries and that CSF does not contain such a large amount of high-abundant protein, such as blood/plasma [20]. The extraction protocol used provided approx. 900 μg ± 350 (SD) proteins from approx. 4 ml of each CSF sample, which enabled us to perform at least three technical 2-DE map replicates for each patient. The average total protein concentration was 24.2 mg/dl ± 9,5 (SD) (range 11 to 43 mg/dl). A mean of 438 ± 94 SD spots were detected per single 2-DE map and approx. 80% of all the detected spots were matched with the respective technical replicates. The variability of technical replicates among matched spots was evaluated and expressed as a mean percentage SD (%SD): this value ranged from 17% to 32%. Replicates showing a variability higher than 32% were excluded from the analysis and new replicates were produced: the two best replicates were kept and used for the final analysis. All the selected 2-DE gels were included in a single class in the workspace of the Image Master software package and matched with the selected reference gel. The matching rate of each map versus the selected reference gel ranged from 65% to 97%.

### Cluster analysis and clinical correlation

**Fig 2:**
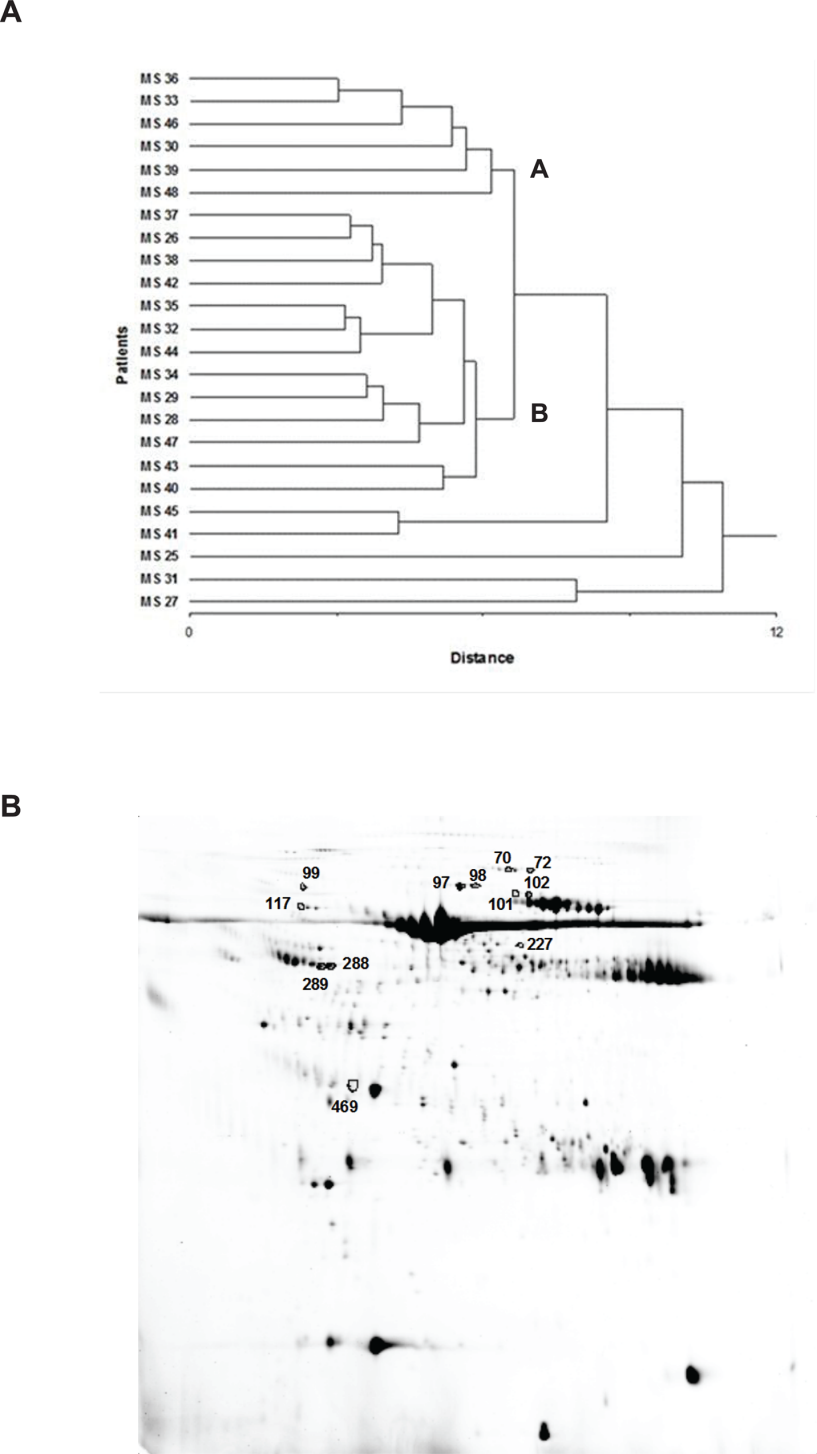
Cluster analysis based on 2-DE data from CSF gels and differently expressed spots. (A) Hierarchical cluster analysis based on the %Vol values of the 239 most representative spots of the analysed gel population. For each spot, the mean value of %Vol was calculated considering the best two replicates (average SD lower than 30%) among 2-DE maps performed in triplicates. Two main clusters (A and B) and five outliers were identified. (B) A representative 2-DE map of CSF: differently expressed spots between cluster A and B are outlined in black.

The results were expressed as the mean value of %Vol ± SD calculated considering the best two replicates among the 2-DE maps performed in triplicates. A clustering analysis, performed as described in Materials and Methods, was carried out selecting the most representative spots present in the entire gel population (239 spots). Two main clusters (A and B) and five outliers were identified with this approach (Fig 2A).

Clusters A and B did not differ by patient age, total protein and albumin concentration in either CSF or serum, Ig Index or CSF cell number (Fig 1A–1D, and Table 2).

Among the five outliers, MS27, MS31 and MS25 represented the patients who had the highest (MS27 and MS31) and the lowest (MS25) amount of CSF immunoglobulin G (IgG), respectively, while patients MS41 and MS45 showed negative oligoclonal bands (OCBs). The diverse amount of IgG in the three patients was also confirmed by the different %Vol of the spots corresponding to light chains of IgG in the 2-DE maps (Fig 1E). Patient MS25, who is the oldest woman participating in the study, has recently been declared as Primary Progressive MS (PPMS).

In order to determine the most relevant spots for distinguishing between clusters A and B, a MannWhitney’s test (threshold: P<0.05, performed using GraphPad Instat version 3.00 software) on %Vol values was applied (Table 3).

**Table 3:** Statistical analysis of spots differently expressed between cluster A and cluster B.

Spot ID	Cluster A (%Vol)	Cluster B (%Vol)	P Value (<0.05)	Fold-change§ (>1.5)
70	0.045 ± 0.008	0.034 ± 0.008	*	~
72	0.040 ± 0.008	0.024 ± 0.014	**	↓1.7
97	0.125 ± 0.035	0.210 ± 0.057	**	↑1.7
98	0.033 ± 0.003	0.046 ± 0.011	*	~
99	0.022 ± 0.007	0.015 ± 0.005	*	↓ 1.5
101	0.053 ± 0.012	0.041 ± 0.006	*	~
102	0.108 ± 0.023	0.082 ± 0.013	*	~
117	0.062 ± 0.015	0.048 ± 0.014	*	~
227	0.034 ± 0.009	0.026 ± 0.005	*	~
288	0.224 ± 0.150	0.139 ± 0.099	NS	↓1.6
289	0.121 ± 0.025	0.214 ± 0.082	*	↑1.7
469	0.123 ± 0.048	0.190 ± 0.0944	NS	↑1.5

§ In the fold change column cluster B is compared to cluster A. Values are expressed as the mean of Vol% ± SD of spots detected in 2-DE map from each patient included in cluster A or B.

NS = not statistical significant (Mann Whitney test).

By using this statistical approach, a panel of 12 differently expressed spots (Fig 2B) were found which were subsequently identified with mass spectrometry analysis (Table 4).

**Table 4:** Mass spectrometry identification of protein differently expressed between cluster A and B.

Spot ID	LC-MS/MS identification	Accession number (gi)	Peptide number	Aminoacidic coverage (%)	Sequence coverage	Theoretical MW (KDa)/pI	Mascot score
**70**	Complement B factor	291922	10	18	51–696	85.4/6.5	206
**72**	Complement B factor	291922	12	18	183–707	85.4/6.5	221
**97**	Gelsolin isoform precursor	4504165	20	36	33–748	85.6/5.9	416
**98**	Gelsolin isoform precursor	4504165	6	10	148–738	85.6/5.9	117
**99**	Afamin precursor	4501987	6	11	89–453	69/5.6	62
**101**	Serum transferrin	110590597	5	10	25–508	74.6/6.6	158
**102**	Serum transferrin	110590597	19	29	25–674	74.6/6.6	342
**117**	Alpha 1-b-glycoprotein	69990	3	6	86–415	51.9/5.6	77
**227**	Albumin fragment	28592	3	5	427–581	69.3/6	54
**288**	Vitamin D-binding protein	181482	12	29	52–440	53.0/5.4	277
**289**	Vitamin D-binding protein	181482	20	56	52–471	52.9/5.4	494
**469**	Apolipoprotein E	178853	18	63	20–317	36.1/5.8	424

**Fig 3:**
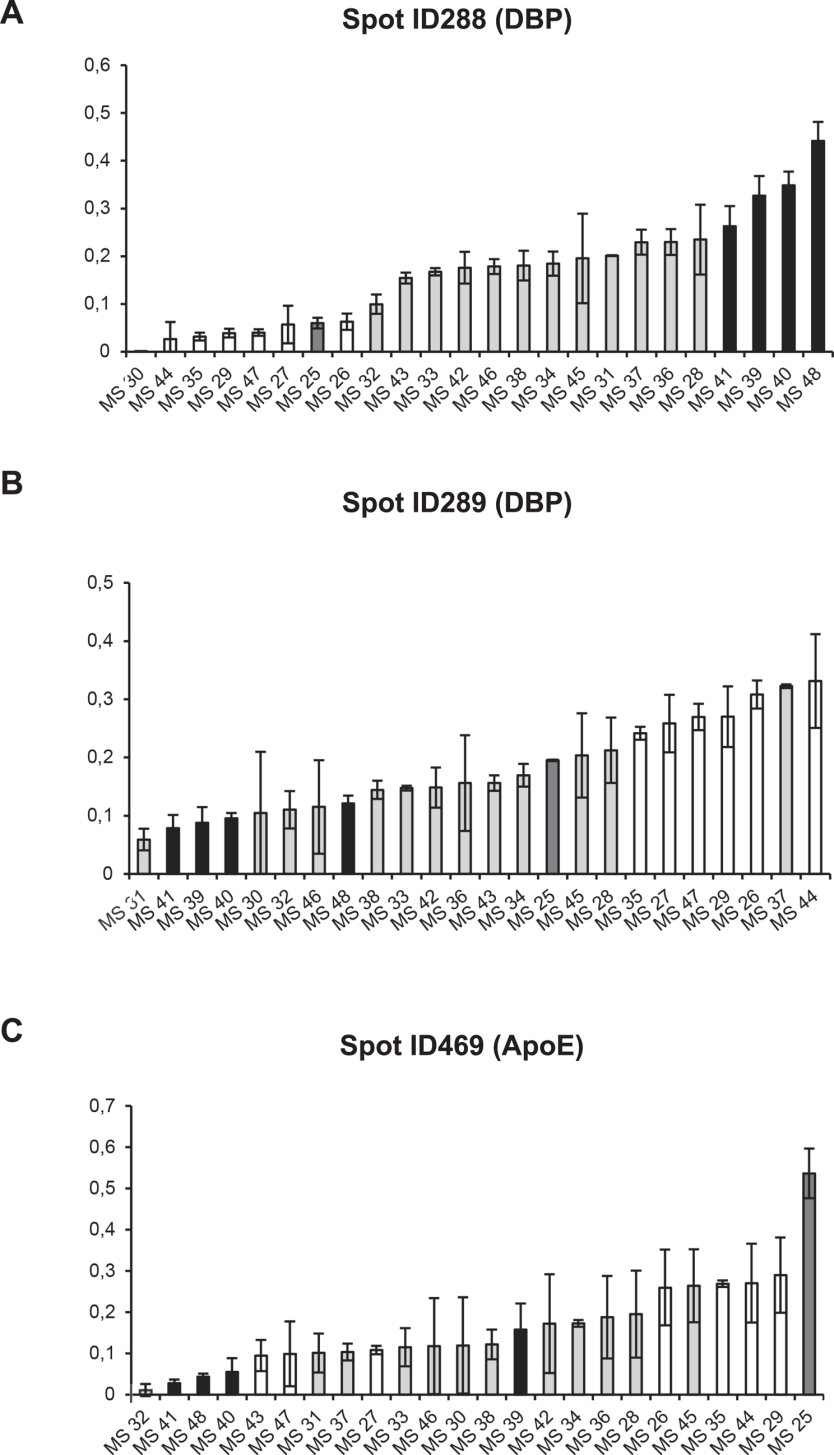
%Vol of spots ID288, ID289 and ID469 in individual patients. Graphical representation of %Vol values of spots ID288, ID289 and ID469 showing the widest inter-individual range in the population studied (0,00–0,44; 0,06–0,33; 0,01–0,54 respectively). The %Vol values are expressed as the mean of at least three replicates ± SD.

Among these, three spots showing the widest inter-individual %Vol variation within the analysed samples were selected, namely spots ID288, ID289 and ID469 (0.00–0.44; 0.06–0.33; 0.01–0.54 respectively) (Fig 3). They were identified as follows: spot ID469 as ApoE; spots ID288 and ID289 as two isoforms of DBP (Table 4). We therefore focused on these two proteins and the three corresponding spots in order to perform a further cluster analyses.

#### Two spot-based (ID288, 289) cluster analysis (DBP isoforms)

The combination of spot ID289 and ID288%Vol values in the following two linear functions, obtained by means of linear discriminant analysis

f1=33.813*(%VolspotID288)+48.942*(%VolspotID289)−7.155

**Fig 4:**
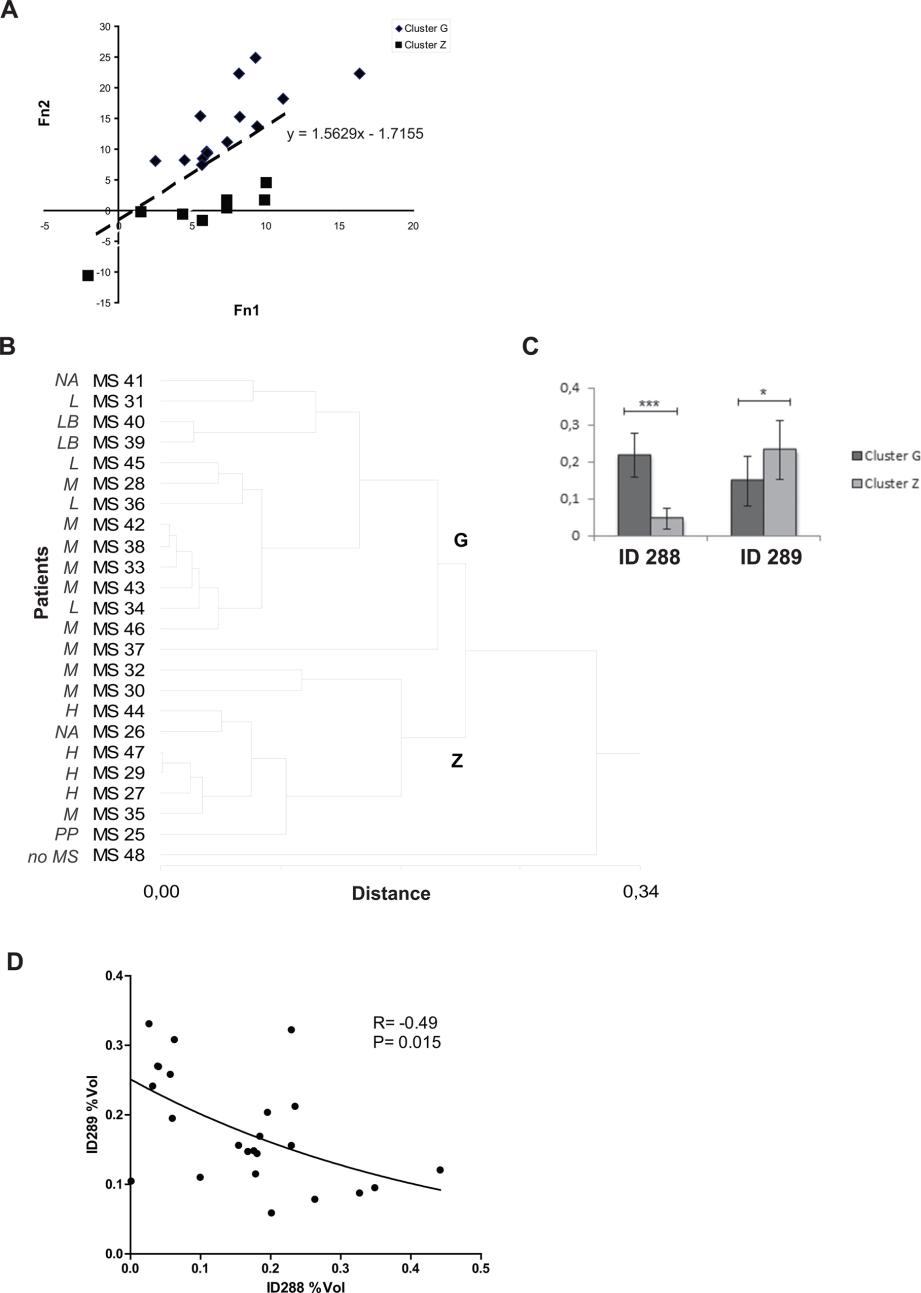
Two spot-based cluster analysis (spot ID 288 e ID 289). (A) Graph showing the results of linear discriminant analysis based on the two linear functions (f1 = 33.813*(%Vol spot ID288) + 48.942*(%Vol spot ID289)– 7.155; f2 = 103.508*(%Vol spot ID288) + 42.871*(%Vol spot ID289)– 15.248) allowing optimal separation of clusters G and Z based on the measured variables (spot ID 288 and ID 289). The mathematical function separating the two groups is also reported. (B) Hierarchical cluster analysis based on the %Vol values of spots ID288 and ID289 identify two clusters, G and Z, and one outlier MS48. The clinical classification of the patients included in the study is indicated alongside the sample number: L = MS patients with a mildly aggressive disease; LB = MS patients with a benign form of the disease; M = MS patients with a moderately aggressive disease; H = MS patients with a highly aggressive disease; PP = MS patients with a primary progressive form of the disease; NA: patients lost to follow-up; no MS: patient with an unconfirmed diagnosis of MS. (C) Column bar graph shows %Vol values of spots ID288 and ID289 in clusters Z and G: values are expressed as the mean of three technical replicates ± SD (bars). * *P*<0.05, *** *P*<0.001 (Mann Whitney’s Test). (D) Spearman statistical analysis shows a significant inverse correlation between spots ID288 and ID289%Vol values for each subject (r = -0.49, p = 0.015).

f2=103.508*(%VolspotID288)+42.871*(%VolspotID289)−15.248

enabled us to divide the entire population into two groups (Fig 4A). This result, also represented by a dendrogram obtained by carrying out a cluster analysis on these two spots, led to the identification of two new clusters (G and Z) and an outlier (MS48) (Fig 4B).

The column bar graphs reported in Fig 4C show an inverse trend of the average %Vol values of spot ID288 and ID289 in clusters G and Z.

An opposite trend of the two DBP isoforms in the entire population had already been highlighted by the content of the corresponding two spots in single CSF samples (Fig 3A and 3B). In fact, most patients with the highest values of spot ID288 showed the lowest values of spot ID289 and vice versa. The inverse distribution of the %Vol of spots ID288 and ID289 in the sample population was also confirmed by the Spearman correlation test (r = -0.49, p = 0.015, Fig 4D) which showed that the spot values in each patient were inversely correlated with one another.

Cluster analysis was therefore correlated with the clinical classification (Table 5) according to the disease-modifying treatment at 2- and 5-year follow-ups (Tables 1 and 5).

**Table 5:** Patient categorization according to two and three spot-based clustering and disease aggressiveness.

**Patients**	**Cluster (288, 289)**	**Cluster (288, 289, 469)**	**Clinical classification 2 years fu**	**Clinical classification 5 years fu**
**MS25**	**Z**	**OUT**	**PP**	**PP**
**MS26**	**Z**	**B1**	**M**	**death**
**MS27**	**Z**	**B1**	**H**	**H**
**MS29**	**Z**	**B1**	**H**	**H**
**MS44**	**Z**	**B1**	**H**	**H**
**MS47**	**Z**	**B1**	**H**	**H**
**MS28**	**G**	**D1**	**M**	**H**
**MS35**	**Z**	**B1**	**M**	**M**
**MS30**	**Z**	**D1**	**M**	**M**
**MS31**	**G**	**D1**	**L**	**M**
**MS32**	**Z**	**D1**	**M**	**M**
**MS33**	**G**	**D1**	**M**	**M**
**MS37**	**G**	**D1**	**M**	**M**
**MS38**	**G**	**D1**	**M**	**M**
**MS42**	**G**	**D1**	**M**	**M**
**MS43**	**G**	**D1**	**M**	**M**
**MS46**	**G**	**D1**	**M**	**M**
**MS34**	**G**	**D1**	**L**	**L**
**MS36**	**G**	**D1**	**L**	**L**
**MS45**	**G**	**D1**	**L**	**L**
**MS39**	**G**	**C1**	**L (B)**	**L (B)**
**MS40**	**G**	**C1**	**L (B)**	**L (B)**
**MS41**	**G**	**C1**	**lost**	**lost**
**MS48**	**OUT**	**C1**	**no MS**	**no MS**

H (high) = highly aggressive disease form, second-line approved therapies (natalizumab or fingolimod); M (moderate) = moderately aggressive disease, first-line approved therapies (interferon beta or glatiramer acetate); L (low) = scarcely aggressive or L (B) “benign” disease, no therapy. The “benign” forms of MS are characterized by no disease progression/activity without specific disease-modifying treatments for more than 10 years. Patients are ranked based on the clinical category to 5 years follow-up (fu). Fu = follow-up; OUT = outlier; lost = patients lost at follow-up.

Notably, the two-spot based cluster analysis showed that most patients suffering from aggressive disease (H category) were clustered together. In fact, cluster Z included all 4 patients belonging to the H subgroup (100%) after the 2-year follow-up, 4 out of 5 patients (80%) belonging to that subgroup after the 5-year follow-up (Tables 5 and 6) and patient MS25 diagnosed with PPMS.

**Table 6:** Descriptive statistical analysis of H, M, L patient distribution in G, Z, B1, C1, D1 clusters at 2 and 5 years follow-up.

**2 YEARS FU**	**Clinical classification**		
	**H**	**L**	**M**
**2 cluster identification**			
**G**	0%	83.33%	72.73%
**Z**	100%	16.67%	27.27%
**3 cluster identification**			
**B1**	100%	16.67%	9.09%
**C1**	0%	33.33%	0%
**D1**	0%	50%	90.91%
**5 YEARS FU**			
	**H**	**L**	**M**
**2 cluster identification**			
**G**	20%	100%	70%
**Z**	80%	0%	30%
**3 cluster identification**			
**B1**	80%	0%	10%
**C1**	0%	40%	0%
**D1**	20%	60%	90%

FU = follow-up.

On the other hand, Cluster G included 70% of patients with moderate (M) forms at the 5-year follow-up and all patients characterized by very slow disease progression (L), also including the two patients defined as “benign” (L-B) MS (MS39 and MS40) (see Material and Methods) (Tables 5 and 6). Interestingly, the final diagnosis of the outlier (MS48) was not MS but somatoform disorder.

#### Three spot-based (ID288, 289, 469) cluster analysis (DBP isoforms and ApoE)

A further cluster analysis was performed which also included spot ID469 in order to assess the ApoE contribution (spot ID469) to patient stratification obtained considering the DBP isoforms (spots ID288 and ID289).

**Fig 5:**
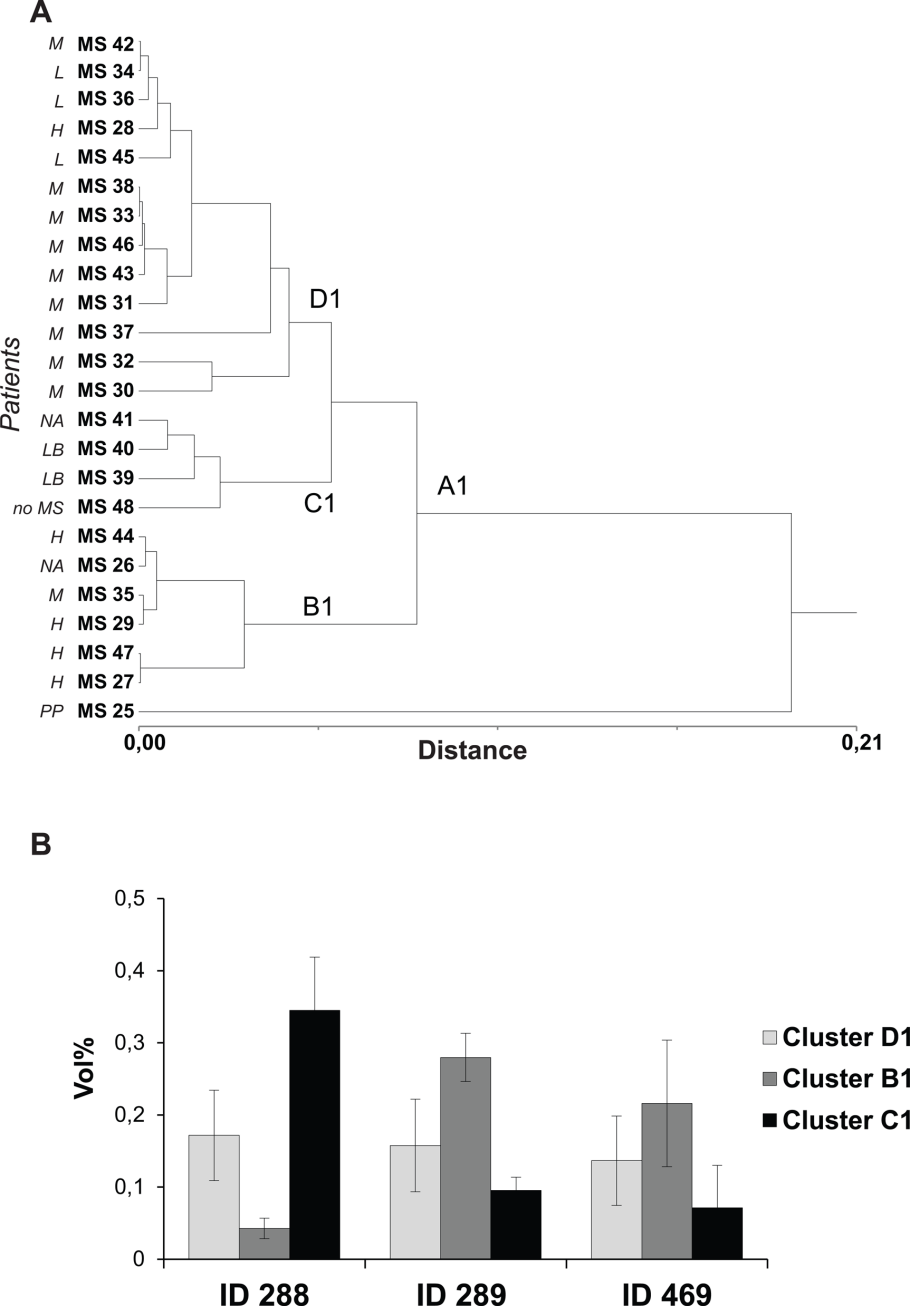
Three spot-based cluster analysis (ID 288, ID 289 and ID469). (A) Dendrogram of the hierarchical cluster analysis based on the %Vol values of spots ID288, ID289 (DBP isoforms) and ID469 (ApoE), showing wider inter-individual range of %Vol values in the differently expressed spots. (B) Column bar graph showing %Vol values of spots ID 288, ID 289, ID 469 in clusters B1, C1 and D1. Values are expressed as the mean of the technical replicates ± SD (bars).

The three spot-based clustering highlighted 3 subsets of patients (B1, C1, D1) (Fig 5A) and singled out subject MS25 as an outlier: this patient was later diagnosed as having a PPMS form.

Spots ID469 and ID289 showed the highest mean %Vol in cluster B1 and the lowest mean %Vol in C1 (Fig 5B). On the contrary, spot ID288 once again showed an opposite trend compared to spot ID289 with the highest %Vol average in cluster C1 and the lowest %Vol average in B1 (Fig 5B).

Considering the clinical evaluations, cluster B1 and D1 were very similar to cluster Z and G respectively, while the addition of ApoE to patient stratification enabled us to isolate a small group of patients included in cluster C1 (Table 5).

This cluster contained 4 patients. Follow-up data were available for 3 of them while one patient (MS41), lacking follow-up data, had a 13-year disease duration before LP and she was discharged without undergoing any treatment. Two patients (MS39 and MS40) included in the L category were the only participants with “benign” MS (Tables 1 and 5) who had a long disease duration (33 and 19 years, respectively), EDSS at the LP time of 1.5 and 1.0 respectively and a ΔEDSS after 5 years equal to zero; they had never taken any DMT. As already mentioned, the fourth subject (MS 48) was suffering from a somatoform disorder.

On the other hand, similar to cluster Z, cluster B1 included 4 out of 5 patients (80%) with highly aggressive disease (H category) and only 1 out of 10 (10%) patients with a moderate (M) form (Tables 5 and 6). It also included all the patients with an aggressive form after the 2-year follow-up.

The largest cluster D1 was very similar to cluster G and contained 13 patients: 9 out of 10 patients (90%) on a first-line therapy with a moderately aggressive form (M category) of MS, 3 out of 5 patients (60%) with a mildly aggressive disease i)(L) and one patient (MS28) who switched from the M to the H category after 2 years (Tables 5 and 6). Patients with the most aggressive form of MS (cluster B1) showed the lowest amount of spot ID288 and the highest amount of spot ID289. An opposite trend was observed in the “benign” form of MS (cluster C1, Fig 5).

Assuming that the sampling is representative of the prevalence of the various forms of the disease in the population, the probabilities of being classified as H, M or L are reported in Table 7. According to these data, both clusterization at two and three spots (Figs 4B and 5A) showed a statistically significant different distribution of the various forms in the subgroups (p = 0.016 and p = 0.001 respectively, at the two-year follow-up; p = 0.027 and p = 0.002 respectively, at the five-year follow up) (Table 7). However, the three spot-based clustering at the five-year follow-up better identified patients who had developed aggressive and benign forms of MS.

**Table 7:** Probability of being classified as H, M or L by three spot-based (DBP isoforms and ApoE) and two spot-based (DBP isoforms) clustering at 2 and 5 years follow-up.

**p-value**	**2 YEARS FU**	**Clinical classification**		
	**2 cluster identification**	**H**	**L**	**M**
0.016	**G**	0%	38.28%	61.72%
	**Z**	50.06%	12.77%	37.17%
	**3 cluster identification**			
0.001	**B1**	66.56%	16.97%	16.47%
	**C1**	0%	100%	0%
	**D1**	0%	23.06%	76.94%
	**5 YEARS FU**			
	**2 cluster identification**	**H**	**L**	**M**
0.027	**G**	7.69%	38.46%	53.84%
	**Z**	57.14%	0%	42.86%
	**3 cluster identification**			
0.002	**B1**	80%	0%	20%
	**C1**	0%	100%	0%
	**D1**	7.69%	23.08%	69.23%

FU = follow-up.

### 2-DE western blotting validation of the different distribution of ApoE and DBP isoforms in aggressive and benign MS patients

**Fig 6:**
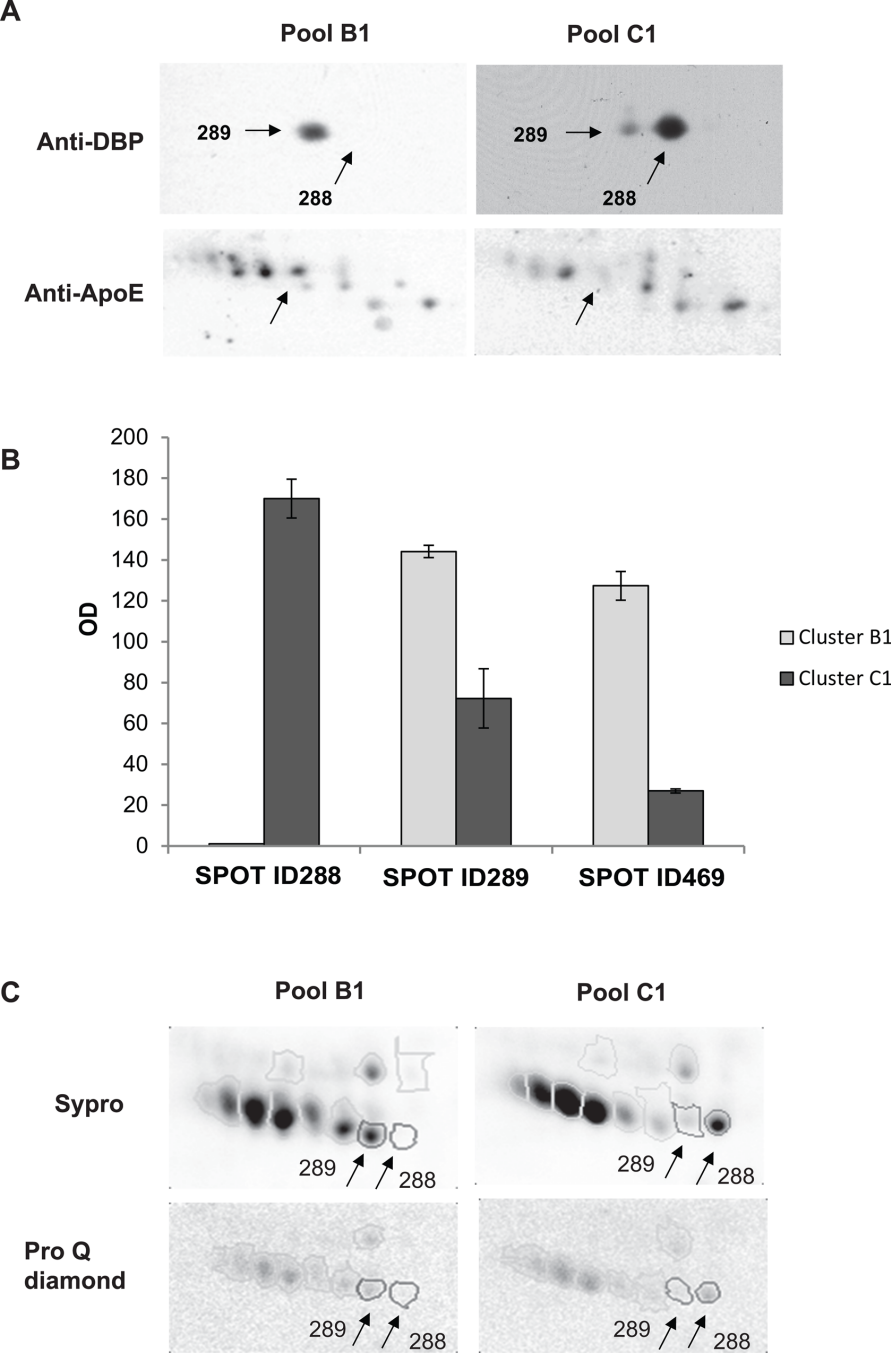
Validation of 2-DE results by western blotting and phospho-proteomic analyses. (A) Representative 2-DE western blotting of DBP and ApoE obtained from pooled CSF samples from patients included in clusters B1 and C1. Arrows point to DBP isoforms (spots ID288 and ID289) in the upper panel, and to ApoE in the lower one. (B) Column bar graph showing optical density (OD) values of the spots visualized by 2-DE immunoblotting (ID288, ID289 and ID469). Values are expressed as the mean of three technical replicates ± SD (bars). (C) ProQ Diamond- and Sypro-stained 2-DE gels obtained from pooled CSF samples from patients included in clusters B1 with that of C1.

2-DE Western blotting on pooled samples with specific antibodies against ApoE and DBP confirmed the %Vol difference between clusters C1 and B1 (Fig 6A and 6B), which mainly included benign and aggressive forms, respectively.

### Phospho-proteomic analyses of DBP isoforms

Post-translational modification (PTM) of DBP isoforms, i.e. phosphorylation, has already been described and a possible role in MS has been suggested [21]. In order to verify whether phosphorylation could be responsible for the two DBP isoforms, we carried out preliminary phospho-proteomic experiments using the ProQ Diamond staining technology and comparing the pooled CSF of patients in cluster B1 with that of cluster C1. The ratio between ProQ and Sypro intensity signal (D/S) of spots ID288 and ID289 was lower than 0.08, meaning that these proteins are not phosphorylated (manufacturer’s instructions, Invitrogen, Eugene, OR). This suggests other kinds of PTM, for example glycosylation (Fig 6C).

## Discussion

MS is a neurological disease characterized by a complex pathogenesis and an unpredictable prognosis. Over the years, it has become evident that the processes concerning the clinical manifestations of MS such as inflammation, demyelination, axonal damage and repair mechanisms, are not equally observed in all patients but can electively predominate in individual subjects. This implies that there are many expressions of the disease and various types of response to treatment [1].

Most MS lesions are typically located in the periventricular white matter of the brain and in superficial areas of the spinal cord, which share anatomical proximity to the liquoral space. As brain biopsies are generally unavailable, the CSF represents a unique repository of pathological information, since it is secreted from CNS structures and contains peptides, proteolytic fragments and antibodies, which may reflect the presence and progression of the disease [22].

To date, despite extensive literature on CSF biomarkers in MS, only qualitative and quantitative biochemical methods are used in clinical practice for assessing the intrathecal production of immunoglobulins [23].

It is essential to find prognostic biomarkers in the CSF in order to enable researchers and clinicians to prescribe appropriate and personalized therapies.

Innovative ‘omics’ techniques are currently available for carrying out research on biomarkers at various levels of expression (genomic, transcriptomic, proteomic and metabolomic). In particular, proteomic technologies such as immunoblotting, isoelectric focusing, 2-DE and mass spectrometry, have proved to be useful tools for identifying the biomolecules required for categorizing the various subtypes of diseases.

Up to now, CSF proteomic data generated by various laboratories are still extremely variable and have not yet been validated [24]. This is partly due to the intrinsic variability of the technique, but it is also influenced by pre-analytical factors (i.e. sample collection, patient heterogeneity, poor experimental design, blood contamination, processing and storage artifacts).

Moreover, considering the limited amounts of CSF collected, most proteomic studies were performed on pooled CSF samples. Although the pooling strategy may minimize potential inter-individual differences of CSF protein content that are not related to the disease, CSF pooling may hinder the detection of markers of the various subtypes of the disease with potentially different pathological mechanisms [10]. Therefore, it is preferable to analyse individual CSF.

In this study we followed precise inclusion criteria for selecting the patients and CSF samples and respected standardized procedures during sampling, handling and storage phases and during 2-DE analyses. Thanks to the availability of a large amount of CSF obtained from each patient by means of an innovative LP protocol [14], we were able to perform 2-DE analysis on individual CSF samples with an adequate number of replicates (at least three with less than 30% variability). All 2-DE analyses were carried out by the same operators, under standardized conditions over a short period of time.

A control group was not included in the study since the goal was to identify CSF biological markers that are able to predict the prognosis of MS. Moreover, it is difficult to obtain CSF from healthy controls and the criteria for classifying the various control subsets have only recently been established [5]. Interestingly, the only patient diagnosed with somatophorm disorder was classified as an outlier in the clusterization concerning DBP isoforms (Fig 4B) and she was included in the subset of patients suffering from less aggressive MS when ApoE and DBP isoforms were considered together (Fig 5A, Table 5).

EDSS and relapse rates are the traditional clinical parameters used for measuring the progression and aggressiveness of MS in natural history. However, the availability of DMTs, in particular NAT and FIN, have greatly reduced the relapse rate and disability progression [25, 26] of RRMS forms, making these parameters a measure of responsiveness to the drugs and not of disease aggressiveness. The aim of this study was to explore markers of disease aggressiveness defined by the type of DMT proposed or prescribed to the patients at the end of the follow-up.

The hierarchical clustering analysis of the 239 spots obtained with the 2-DE analysis, a non-hypothesis driven approach, initially selected 12 spots (Fig 2B and Table 4).

Further cluster analysis of the 12 spots showed that three of them, two isoforms of DBP (spot ID288 and ID289) and an isoform of ApoE (spot ID469), were the key proteins responsible for driving the clustering. The correlation between the subsets of patients obtained by means of the cluster analysis and the subsets of patients classified according to the degree of disease aggressiveness (H, M and L) showed that this approach can help to predict the clinical course. In particular, patient stratification obtained with the cluster analysis according to the two isoforms of DBP identified patients who were likely to develop a highly aggressive form (H category) of MS in the following 5 years (cluster Z, Fig 4B and Table 5). Adding ApoE to the cluster analysis improved the categorization of patients (Fig 5A and Table 5). In fact, the three spot-based clustering approach correctly grouped 4 out of the 5 patients with aggressive MS in cluster B1 at the 5-year follow-up and identified 9 out of the 10 patients suffering from a moderate form in the cluster D1. By clustering with DBP isoforms and ApoE, it was also able to identify a small group of patients (cluster C1) characterized by a”benign” form (L-B category) or with no disease in one case (MS48). As shown in Table 7, the probability of being classified as H, M or L differs significantly among the clusters obtained with both approaches (two spot- and three spot-based clusterization). The three spot-based analysis better correlated with the classification based on the clinical course at the five-year follow-up. In fact, there is an 80% probability that the disease will evolve into aggressive forms for the patients in cluster B1, while there is a 100% probability that the disease will remain “benign” for the patients in cluster C1 (Table 7).

Further investigation revealed that the amounts of the two DBP isoforms (spot ID288 and ID289) did not change concurrently but they showed an inverse correlation: by describing the combination of the %Vol of these two spots by means of two linear functions, it is possible to separate highly aggressive MS forms from moderate/low MS forms.

Besides transporting vitamin D metabolites [27], DBP carries out several important functions, including actin sequestration and a range of less-defined roles in modulating immune and inflammatory responses. Several studies suggest a role of DBP in MS [6, 7, 8, 12, 28, 29]. Considering the association between low vitamin D status and MS [30], many have searched for a possible association between the concentration of DBP in CSF or serum and MS with equivocal results [6, 12, 24, 31, 32, 33]. In our study, the inverse correlation of the two DBP isoforms suggested the relative prevalence of one of the isoforms rather than the total DBP accounts for the patient categorization. This suggests that a PTM may be related to the degree of aggressiveness and therefore have a prognostic meaning.

DBP may undergo various kinds of PTM, among which phosphorylation and glycosylation. Erikson *et al*. [21] proposed the level of phosphorylated DBP in CSF as a promising tool for distinguishing relapsing-remitting MS from secondary progressive MS forms and from other neurological diseases. Our preliminary phospho-proteomic experiments did not confirm a difference in DBP phosphorylation status (Fig 6C).

Glycosylation is another PTM which may explain the presence of two DBP isoforms in 2-DE gels. In fact, DBP occurs in several isoforms with various levels of glycosylation [34, 35, 36]. Different levels of glycosylated (sialylated) isoforms of DBP with predictive clinical value had already been demonstrated in a 2-DE study carried out on juvenile idiopathic arthritis [37], a poorly understood group of chronic autoimmune diseases.

The degree of glycosylation seems to regulate the inflammatory and immunological activities of DBP. Glycosylation of DBP through the combined action of a betagalactosidase and a sialidase engenders the Gc macrophage activating factor (GcMAF), the most potent activator of macrophages with an important role in immune system regulation and anti-angiogenetic activity in cancer patients [34, 38]. The two spots found in this study might differ for their glycosylation status. As the glycosylation of DBP isoforms affects the binding affinity for vitamin D metabolites and consequently their circulating levels [39, 40, 41], we can speculate that the degree of DBP glycosylation or the activity of glycosylating/deglycosylating enzymes may have a prognostic and diagnostic value in MS. Glycoproteomic experiments aimed at verifying this hypothesis are in progress in our laboratories.

The second protein which contributed to the identification of the clinically relevant subtypes is ApoE (spot ID469). Many researchers have reported the association between ApoE ε4 allele and the increased risk of MS, showing that ε4 predisposes to faster progression [42]. The 2-DE analysis did not enable us to distinguish among the different ApoE allelic forms. However, the patients included in cluster C1, characterized by a benign course, had lower levels of ApoE than those in cluster B1, who developed highly aggressive forms.

Although there are some limitations in the study (i.e. small number of patients, the use of semi-quantitative method), the non-hypothesis driven approach combined with accurate pre-analitical procedures, standardized laboratory methods and 5-year follow-ups of women with suspected MS, enabled us to identify potential biomarkers of MS prognosis. In addition, these biomarkers could also have a biological role in the pathogenesis of the disease. Obviously, these findings require wider validation studies to be carried out on a larger population in various laboratories.

## Conclusions and Perspectives

This study proposes a novel biomolecular tool consisting of two isoforms of DBP and ApoE which may be useful for monitoring the progression of the disease. If validated on an larger independent population of retrospectively selected CSF samples, this study could positively effect the treatment, prognosis and understanding of MS and assist the clinician(s) in the daily management of MS patients. Future activities will involve the analysis of a control group including other neurological patients in order to confirm the specificity of the results for MS.

Further studies on the glycosylation status of DBP and the activity of the enzymes involved in glycosylation/deglycosylation may shed light on the pathogenesis of the disease and help determine new goals for treatment.

The use of DBP as a prognostic tool in MS has been patented (EP2664923).

## Supporting Information

S1 Dataset 2-DE gels dataset. %Vol values of the most representative spots (239 spots): in sheet #1 mean values of spots and hierarchical clustering analysis performed by StatisticXL are reported; in sheet #2% Vol values, of each replicate, mean value and standard deviation are reported.
(XLS)

S2 Dataset Dataset from 2-DE replicates. The zipped folder includes the 24 files related to the 2_DE dataset from CSF sample maps of each patient included in the study. In particular, in each file, %Vol values of matched spots between replicates are reported. For each spot mean and standard deviation (SD) were calculated and the value of variability (expressed as mean SD percentage, %SD) is reported.
(ZIP)

S3 Dataset Mass spectrometry protein identification. Details of protein identification, as provided by Mascot search algorithm, are reported for each identified spot (peptides assigned to the identified protein are evidenced in bold).
(ZIP)

S1 Images 2-DE gel population images. The zipped folder includes the 48 acquired 2-DE gels submitted to the final image analysis.
(ZIP)
